# Advances in the agent-based modeling of economic and social behavior

**DOI:** 10.1007/s43546-021-00103-3

**Published:** 2021-07-07

**Authors:** Mitja Steinbacher, Matthias Raddant, Fariba Karimi, Eva Camacho Cuena, Simone Alfarano, Giulia Iori, Thomas Lux

**Affiliations:** 1 Department of Mathematics and Statistics, Faculty of Law and Business Studies, Krekov trg 1, 1000 Ljubljana, Slovenia; 2 Department of Economics, Kiel University, Olshausenstraße 40, 24118 Kiel, Germany; 3 Complexity Science Hub Vienna, Josefstädter Str. 39, 1080 Vienna, Austria; 4 Department of Computational Social Science, GESIS, Leibniz Institute for the Social Sciences, Unter Sachsenhausen 6, 50667 Cologne, Germany; 5 Department of Economics, Jaume I University, Avenida Sos Baynat s/n, 12071 Castellón, Spain; 6 Department of Economics, School of Social Science, City University, Northampton Square, London, EC1V 0HB UK

**Keywords:** Agent-based models, Heuristic design, Model calibration, Networks, Behavioral economics, Computational social science, Computational economics, B41, C60, D90, E70, G17, G40, L20

## Abstract

In this review we discuss advances in the agent-based modeling of economic and social systems. We show the state of the art of the heuristic design of agents and how behavioral economics and laboratory experiments have improved the modeling of agent behavior. We further discuss how economic networks and social systems can be modeled and we discuss novel methodology and data sources. Lastly, we present an overview of estimation techniques to calibrate and validate agent-based models and show avenues for future research.

## Introduction

Agent-based models (ABMs^1^) are a way to model and simulate the behavior and interactions of heterogeneous individuals and organizations and to infer regularities that govern their behavior as a whole. While first traces of ABM can be found in the 1950s, ABM as a methodology was only popularized in the 90s when computational methods became more readily available.^2^ By today, agent-based modeling has been applied to a large number of scientific fields and it continues to be an exciting and popular approach for a number of reasons:

1. The availability of computational power to model large-scale social interaction;
2. The possibility to use decision rules to model behavior (behavioral heuristics) instead of mathematical optimization;
3. The increasing popularity of behavioral research in economics that provides insights for designing agent-based models;
4. The rapid development of network theory in the social sciences that provides new tools for the formalization of interactions between agents;
5. The importance of the stability of human-devised systems (such as the financial system);
6. Advances in the estimation and calibration of agent-based models that allow a better assessment of their goodness-of-fit for empirical data.

This survey highlights the above listed concepts and presents applications of modeling economic and social behavior that have seen a significant development in the last decade. Our goal is to provide an overview of the state of the art and explore some of the potentials of the agent-based approach along these lines.

First, in “Agent-based models and computational social science”, we will see how advances in models with (mostly) heterogeneous agents have led to much more detailed simulations of social behavior and social systems and how this has contributed to a better understanding of how agents’ behavior and interactions lead to structure on the aggregate level. A significant part of this section is devoted to the granularity of data and data types that can be used in agent-based models. In “Heuristics and modeling”, we discuss the use of heuristics in defining adaptive behavior of boundedly rational agents such as households, financial investors, banks, and/or firms by sourcing from some of the most recent agent-based models within the fields of economics and finance. In Sect. Economic networks, we present how economic networks can be used to describe the interactions of agents, for example when these represent organizations, such as firms or banks. This section focuses on advances of structure identification in economic networks and brings forth some recent examples of explicit incorporation of networks into agent-based models. In what follows, “Agent-based models and financial stability” highlights one particular case where networks have proven very useful, namely in the analysis of systemic stability of the financial sector. Here, idiosyncratic actions can result in correlated responses leading to aggregate fluctuations and macro-level instabilities. The section presents a compilation of agent-based models that study connectivity within a banking system, emerging systemic risk, and address the risk mitigation via macro-prudential rules (such as leverage ratios, liquidity ratios, equity ratios) and tax policies. “Controlled laboratory experiments” is motivated by the fact that behavioral economics has only recently connected to the literature about computational methods. This section describes the contribution of experimental and behavioral economics to agent-based modeling in dealing with the behavior and interaction of heterogeneous agents. It is focused on the need to combine computational economics with the capacity of controlled laboratory experiments to study the effects of psychological, cognitive, emotional, cultural, and social factors on decision making in order to bring the agent-based models closer to experimental data. Finally, “Estimation of agent-based models” elaborates on the development of estimation methodology for agent-based models. While many agent-based models aim to reproduce certain stylized facts of economic systems, their validation too often stays on a rather rudimentary level. This section, therefore, surveys methods for the empirical validation and estimation of agent-based models and their parameters.

## Agent-based models and computational social science

Computational social science (CSS) is receiving enormous momentum in recent years thanks to the availability of large-scale datasets in various forms and the accessibility of computational platforms to social scientists. Broadly speaking, CSS aims to use computational methods and large-scale data to examine existing social theories, develop new theories, and improve our understanding of human behavior in scale. Despite its broad perspective, CSS in recent years focused heavily on data-driven methodologies (Lazer et al. 2020), and the community of agent-based modelers has been largely neglected. In fact, agent-based modeling combined with data-driven methodologies can be extremely instrumental in deepening our understanding of social behavior and guiding us towards their explanation (Conte and Paolucci 2014). Models allow to examine the macro-level outcomes that arise from social and psychological theories and empirical data can be used to validate the models. This is important because there can be many social or psychological theories for a social phenomenon that result in different behavioral outcomes (Lorenz et al. 2020). ABMs in social science consist of multiple components that can be characterized as follows:

1. *Agents with their perceptions and decision-making capacity* Agents are commonly comprised of individuals or social groups that have a set of complex psychological traits and socio-demographic attributes. These attributes can be fixed or dynamic. Epstein argues that we should consider cognitively plausible agents in ABM (Epstein 2014). An example of such an approach is the work by Sircova and colleagues that used cross-cultural survey analysis combined with discussions in focus groups to assess the big five personality traits in different countries and use that to calibrate the level of cooperation among agents when resources are limited (Sircova et al. 2015).
2. *Environment* Agents are often in an environment where they interact with others and where these interactions impact their action and also the environment itself. In his seminal work, Watts showed that when a norm-adoption mechanism is applied on a social network, the size of the adoption cascade is heavily dependent on the structure of social network, since agents do not interact homogeneously with each other (Watts 2002).
3. *Rules and actions* While interaction between agents can be adjusted by a plausible network, the rules of interaction with other agents and decision making processes are deduced from social and psychological theories or observations. For example, the Granovetter threshold theory—people follow a norm as long as a certain threshold of people in their neighborhood follow it—is often used to study the dynamics of norm adoption in a society (Granovetter 1978).
4. *Macro structure* Macro-level structure emerges as a consequence of the micro-level behavior of the agents over time and the macroscopic outcomes may vary significantly from micro behavior (see Schelling 1971, for an early segregation model). This transition from micro to macro allows ABM to be a powerful explanatory tool. By tuning the parameters on the micro-level, the macro-level effect can be examined.

Depending on the purpose of the model, different levels of granularity and data are needed. Edmonds (2017) categorized the purpose of modeling into seven categories, namely prediction, explanation, description, theoretical exploration, illustration, analogy, and social interaction. ABMs offer a practical approach to assess the validity and risks associated with any of these purposes. Understanding the purposes associated with the ABM in CSS will enable an interdisciplinary team to understand and appreciate the usefulness of the model and assess the validity and the scope of the results in a reliable manner.

ABMs have been developed in great detail to analyze mechanisms relevant to sociology, i.e., social influence, cooperation, social norms, the emergence of conventions and culture, and opinion dynamics, to name a few. While there are good reviews on ABMs in sociology (Bianchi and Squazzoni 2015; Conte and Paolucci 2014) and managerial science (Wall 2016), an overview of data resources that could help modelers to move towards data-driven directions is still lacking. In what follows, we will discuss potential data sources and their use in the development of data-driven ABMs.

*Surveys* The use of publicly available surveys such as the European Social Survey (ESS) is the most common approach for initialization of the models or validations. For example, Åberg and Hedström (2011) used unemployment data combined with socio-demographic information of urban neighborhoods to explain the impact of social influence on youth unemployment. In another example Grow and Van Bavel (2015) use ESS to model the relationship between assortative mating and gender inequality in higher education.

*Digital media* Social media data sets are exceedingly being used by the CSS community to extract information about the ideology and attitude of users and how they shape and evolve over time. For example, sentiment analysis on social media platforms can help infer the users’ political and ideological leaning, which will inform the agent’s cognition and behavioral properties (Waldherr and Wettstein 2019). Analyzing the agents’ actions over time could be harvested to infer behavioral aspects such as opinion dynamics and polarization.

*Social network data* Information on who follows whom or data from friendship networks in online social networks can be used to create more realistic interaction scenarios. This information combined with recent advances in the identification of gender or ethnicity of users from the names or images (Karimi et al. 2016) can be used to identify how different groups of people interact based on their socio-demographic attributes. For example, by accounting for homophily in social interactions based on empirical evidence, one can model the spread and adoption of norms between majority and minority groups more realistically (Kohne et al. 2020).

The timing of social interactions can also significantly influence diffusion processes (Karimi and Holme 2013), and thus, temporal networks are hugely instrumental in building realistic models of social interactions over time for studying dynamical processes such as the spread of information, norms, culture, cooperation, coordination, and innovation diffusion (Holme 2015).

*Crowd-sourced data* Conducting large-scale online surveys and focus groups enables researchers to achieve large-scale data to calibrate ABM models or evaluate the outcomes in a viable manner (Behrend et al. 2011). For example, by asking people about their local neighborhood and their estimate about a prevalence of a certain minority group, one can estimate the perception bias of people based on their social network (Lee et al. 2019) and use this information to model disinformation spreading or mitigation strategies to prevent formation of biases.

*Call data and wearable sensors* Found data such as data on mobile phone calls combined with socio-demographic and location information can be used to model information networks and to explore various dynamical aspects of human society, such as the spread of diseases (Gozzi et al. 2020). In more controlled settings, wearable sensors such as sociopattern sensors can be deployed to infer the communication structure in face-to-face interactions and to study how this impacts the performance of students in schools (Fournet and Barrat 2014).

*Scholarly databases* Large-scale scholarly publications such as the Web of Science or the DBLP database can be used to model how scholars move, find new collaborators, how ideas spread, and how a new field of research emerges (Rosvall and Bergstrom 2008).

*Urban mobility and census data* Publicly available data on urban mobility can be used to model communication and movement of people in space and time, e.g., to study how offenders communicate and move in a city (Rosés et al. 2018). Combining census data, panel data and mobility data could help to better model inequality and racial segregation in cities (Crooks 2010).

## Heuristics and modeling

In this section, we will walk through elementary heuristics in some recent agent-based models in economics and finance. We use the notion of the heuristic as a strategy that ignores part of the information to ease the process of decision making (Gigerenzer and Gaissmaier 2011). An extensive survey of action rules (behavioral heuristics) in agent-based models can be found in Dosi et al. (2020).

Heuristics in financial models have long been centered around learning and adaptation in a multi-agent setting and how this interferes with the financial market as a whole (see for instance LeBaron 2002). Financial agents perform trades in financial assets and interact with each other either directly via social learning processes, or indirectly, via the price mechanism. Anufriev and Hommes (2012) develop heuristics to explain coordination of individual behavior as observed in laboratory financial markets. Agents in financial models range from passive automates without cognitive functions (i.e., zero-intelligence agents) to active data-gathering decision makers with learning capacity (i.e., agents with microfunded rules of behavior, such as in Iori and Porter 2018). Financial agents are still developed as optimizers of some objective (or criteria), such as debt/equity ratio (Fischer and Riedler 2014), utility, profit, or other criteria. Optimization algorithms rely on well-defined objective functions, usually of additive or exponential form, of weighted combinations of the criteria under consideration (An 2012).

Learning in financial models can be based on probabilistic learning (Lux 2009b), where people choose between prospects based upon probabilistic alternatives involving risk, such as in Polach and Kukacka (2019). In addition, “probabilistic” agents with adaptive learning might be constructed, such that they adopt strategies based on relative performance to some benchmark or, alternatively, source from an evolving pool of strategies, formed by a mix of chartist and fundamentalist features (Mandes and Winker 2017) with anchoring (Polach and Kukacka 2019) and herding (Vidal-Tomás and Alfarano 2020). Probabilistic learning has traditionally been implemented in the Bayesian way, while adaptive learning rests upon an evolutionary computation with components of genetic algorithms and artificial neural networks. Heuristics in financial models and institutions are focused on simple rules for modeling the flow of funding between cash providers, dealers, and hedge funds as exemplified by Bookstaber et al. (2018).

A wide variety of behavioral heuristics have been developed for modeling agents in economic settings. For instance, Vallino (2014) applies a simple trial-and-error heuristic on procedural rationality of agents in a public choice setting where agents utilize common pool resources (i.e., forests) by adopting their utilization strategies upon changes they observe in the availability of the resources. These agents are boundedly rational (i.e., they do not optimize their objective functions) and operate as satisficers (Simon 1959) within an endogenous institutional setting. Then, there is a trust game simulation experiment (Gazda et al. 2012) of adaptive agent’s behavior, where agents are placed in an exogenous and static institutional framework. Authors use a set of behavioral components and ad hoc heuristics to define agents’ actions. Both examples are implemented in the highly applicable NetLogo environment.

Delli Gatti et al. (2011) argue in favor of agent-based models with many types of agents with a small set of behaviors for each type. According to the authors, heuristic rules, in principle, push the heterogeneity of ad hoc rules to infinity. The authors further stress that agents, in reality, adopt a small portion of behavioral rules and they do not behave in isolation, but via rules for social interaction (i.e., direct or indirect, local or global) with other agents, through learning and mimicking. As a result, agents regularly reformulate expectations about their future states and decisions, and/or impact others’ preferences and/or available choices.

Gurgone et al. (2018) build on the approach suggested by Delli Gatti et al. (2011). Their model consists of households, firms, banks, a government and a central bank. Relations in the model are implemented by heuristic rules via some binding equations. For instance, households follow a rule of thumb to determine consumption (linear in relation to available resources); firms hire labor in a 4-step heuristic and set their liquidity needs in advance (i.e., demand for loans becomes a Markov process); firms charge mark-up prices for their products defined by mark-up rule based on their market share; wages are adopted rule based, taking into account a linear combination of moving average(s) of inflation and unemployment; relations between government, central bank, banks and firms are determined on financial markets and in the banking sector via heuristic rules for the provision of liquidity, borrowing constraints, repayment and tax collection rules. Banks use a probabilistic approach (i.e., logistic default probability based on borrower’s leverage) to model the risk of their borrowers and they use balance-sheet heuristics to monitor liquidity needs and regulatory requirements (i.e., prudential rules).

EURACE (Holcombe et al. 2013) is a large-scale agent-based model of the European economy including labor markets, industry evolution, and credit markets. The model consists of nine types of agents (firms, households, investment goods producers, malls, banks, clearing houses, government, central bank, and Eurostat) that operate in various interrelated markets with institutional agents who assess economic indicators and transmit this information back to economic agents. Behavioral heuristics in the model refer to movement, communication, work, consumption decisions, learning, investment decisions, and speculation on financial markets. Agents are boundedly rational with limited capacity for information assimilation. They use simple rules and can learn to adapt to a changing economic environment. For instance, firms plan inventories based upon expectations of future sales obtained by regressions on historical sales; labor is hired via a set of search-match heuristics applied on firms and households; pricing of consumption goods is based on simple mark-up rules; consumers purchasing decisions are random and probabilistic in nature driven by purchasing probabilities they attach to different products based on prices; central bank uses simple heuristics and Basel rules (i.e., via a Deferred Settlement System) to provide liquidity that banks need to finance loans to firms, etc.

At any rate, behavioral heuristics, as employed in the ABM literature, underutilize vast possibilities offered by the expectation formation theory. Early empirical studies of the expectation formation were pioneered, e.g., by Nerlove (1956) while an early expectation formation theory dates back to Lachmann (1943) and Baudin (1954). Since then, the expectation formation theory has seen a major evolution in economics that went from rational expectations (Muth 1961), learning (Cyert and DeGroot 1974; DeCanio 1979) to irrational expectations and heterogeneity built around satisficing agents (Simon 1959). Apart from the rare ABMs that explicitly build on expectation formation (e.g., see Carroll 2005; Easaw and Ghoshray 2006; Lanne et al. 2009; Reid 2015; Gerotto and Pellizzari 2018, for some examples), an ample space is yet to be utilized by the ABM in this direction.

Heuristics have a critical impact on the behavior of agents in the model. They need to be carefully implemented such that they capture main behavioral attributes of agents under consideration to facilitate their decision making within a particular institutional setting. Moreover, according to Dosi et al. (2020), heuristics may provide a more accurate and robust tool for modeling action also within an uncertain environment than sophisticated techniques.

## Economic networks

The financial crisis of 2008 has led to a drastic rise in the awareness of the importance of network properties of economic systems. The structure of economic networks plays an important role for the robustness of the global economy, for understanding structural change and shocks, and for identifying conflicts between global efficiency and individual interests (Schweitzer et al. 2009). For ABMs, this means that besides modeling the behavior of agents we have to model realistic networks of interactions where these are relevant for the dynamics of the system. This is not an easy endeavor since this mostly necessitates the use of large-scale data sets, which are only gradually becoming available, together with large-scale simulations. This section, therefore, will to a large extend focus on advances of structure identification in economic networks before pointing to a few agent-based approaches that incorporate network structure explicitly.

Small- to medium-scale social networks have been studied in sociology for a long time and have uncovered basic properties of social interactions (see Freeman 2004, for an overview). Larger-scale systems have, however, only been analyzed after the increase of computing capacity in the 90s, and in fact notable studies from that time included the analysis of the structure of the world wide web (Albert et al. 1999). One application of this new approach was studies on cascades (Watts 2002). In economics, such cascade models, which are very similar to models for epidemics (see, e.g., Eubank et al. 2003), have been augmented for the analysis of contagious effects in financial markets. This part, however, will be discussed in more detail in the next section. Here, we will discuss some recent developments that aim at describing economic networks in general.

By today networks have become accepted as mainstream research topics in economics, as they have been identified as decisive influences on economic growth (Acemoglu et al. 2012; Jackson et al. 2017). Even some textbooks have focused on networks in economics (Jackson 2008; Easley and Kleinberg 2010). Nevertheless, it is necessary to understand that much of today’s research is actually based on previous works in sociology, physics and computer science. For example, networks of firms have been analyzed by Uzzi (1996) and Gulati and Gargiulo (1999) from a sociologist’s perspective. Also, the analysis of corporate boards and firm networks (Kogut and Walker 2001; Raddant and Takahashi 2021) overlaps with research in management science (Devos et al. 2009; Zona et al. 2018), corporate finance (Duchin et al. 2010; Herskovic 2018), and interdisciplinary research in physics and computer science (Battiston and Catanzaro 2004; Vitali et al. 2011).^3^

There are several approaches where known agent-based models have been extended to incorporate network structures between agents explicitly, for example in herding models (Alfarano and Milaković 2009), economic games (Wilhite 2014), or Schelling’s well-known segregation model (Fagiolo et al. 2007b; Schelling 1971). These approaches show under which circumstances network structure influences macroscopic outcomes, yet they do not answer which of the proposed structures we find in reality, how they formed, and how they might develop in the future.

The agent-based approaches to economic networks are also a response to the limitations of traditional macroeconomic models (DSGE) in explaining interaction effects, especially with the financial sector, and crises, in particular of course that of 2008 (LeBaron and Tesfatsion 2008; Dosi et al. 2015; Dosi and Roventini 2019). Hence, when it comes to modeling larger economic systems there are currently two overlapping approaches. On the one side, there are classical ABMs that describe economic systems where the agents’ behavior is mostly calibrated to empirical data, one noticeable example is the model for the European economy by Deissenberg et al. (2008). While many models include a matching of agents in different markets, the resulting network structure of these matches is typically not of major importance (see Dawid and Delli Gatti 2018, for an overview). A recent example that takes network structure into account for some parts is the approach by Poledna et al. (2020).

On the other hand, there are models for specific parts of economic systems which are often completely data driven, for example describing the production network of a country like Japan (Krichene et al. 2019). Further examples are the analysis of world trade (Fagiolo et al. 2009) and sector-based input–output networks (Cerina et al. 2015; Klimek et al. 2019). While for many economic networks data of bilateral flows or exposures are available, some markets have been modeled indirectly via the use of time series data and the derivation of correlation-based networks. An example for the latter is the analysis of the dependencies in financial markets for which many different approaches exist (Musmeci et al. 2015; Tumminello et al. 2005; Raddant and Kenett 2021; Diebold and Yilmaz 2014; Billio et al. 2012)

Arguably, most of these contributions are not ABMs, they are empirical studies on economic networks. This distinction is, however, sometimes superficial. The reason is that when we want to estimate the effects that have led to a particular network structure we typically revert back to simulation based inference of these effects, for example in exponential random graph models or the stochastic actor based approach (Strauss and Ikeda 1990; Wasserman and Pattinson 1996; Snijders 2001). Hence, we estimate which behavior on the level of agents has likely led to an observed outcome with respect to network structure.

Noticeably, there is one specific field of research where the agent-based modeling of agents’ behavior and connectivity is mostly done jointly, namely in describing the relationships of firms with financial institutions. While the analysis for the case of Italy (De Masi and Gallegati 2011) is still mostly an empirical study, there are more elaborated models inspired by the stylized facts of loan networks of countries like Italy and Spain (Lux 2016) and an explicit agent-based model for the case of Japan (Bargigli et al. 2014, 2020) where network structure becomes one of the key calibration targets.

## Agent-based models and financial stability

The financial system is a classic example of a complex system. Its dynamic is difficult to predict due to the interconnectedness and interdependence of its parts which give rise to nonlinearities, tipping points, adaptation and feedback loops, among other features. Many empirical financial phenomena, such as fat tailed return distributions, booms and bursts cycles in asset price, volatility clustering, runs on funding, asset fire sales, and financial crises are difficult to explain by traditional economic models based on the conjecture that the actions of fully rational agents are driven by market fundamentals. ABMs instead are built on the assumption that agents are boundedly rational, interacting and heterogenous. Agents idiosyncratic actions can become coordinated, either via direct reciprocal interactions or by indirect reaction to common signals, and lead to large aggregate fluctuations and macro level instabilities. By simulating how banks, investors, regulators, and other players interact with each other, and with the real economy, ABMs have been instrumental in gaining a deeper understanding of how extreme events in real-world financial markets can arise.

Earlier ABM work has focused predominantly on the role of the micro-structure of exchanges (execution policies, order types, execution fees, etc.), market transparency, and the interaction among heterogeneous strategies, on the volatility of stock prices and the dynamics of order flows. ABMs simulations have shown that stock market models do not generally select the rational, fundamentalist strategy and that simple technical trading rules, such as chartist strategies, as well as herding behavior, may survive. These direct and indirect interactions, by acting as a coordination device of agents’ trading decisions, can lead to wild fluctuations in asset prices and memory effects in order flows.

ABMs have been helpful not only to identify the mechanisms that lead to instabilities in financial markets, but also to evaluate policies designed to mitigate them. Pellizzari and Westerhoff (2009), for example, have studied the effect of transaction taxes in an agent-based model in which central dealership or continuous double auction are used as a clearing mechanism. Their work shows that in the former case, the volatility of the market can be significantly reduced via the imposition of a transaction tax; however in the second setting, the tax would reduce market liquidity neutralizing any improvement in price stability. Ladley et al. (2015) have shown that centralizing markets can lead to higher price volatility and less resilience to shocks because it increases the equilibrium proportion of unskilled traders. Kovaleva and Iori (2015) have studied the effects of pre-trade quote transparency on market quality in an artificial limit order market where traders react to the imbalance in demand and supply posted in the limit order book. Their simulations show that full quote transparency leads to high transaction costs that dampen trading volume. While the exogenous restrictions of displayed depth do not improve market quality, endogenous restrictions by means of iceberg orders are effective in balancing the limit order book, reducing transaction costs, maintaining higher liquidity, low volatility, and overall enhancing price discovery.

In recent years, a large part of the ABM financial literature has shifted to the study of systemic risk and in particular to the analysis of the extent to which default cascades are affected by the connectivity among banks. The inter-bank credit market is an important means through which commercial banks cover short-falls in liquidity. By borrowing from banks with surplus liquidity, banks which face a temporary shortfall can survive as a result of inter-bank credit. This represents risk-sharing and, in and of itself, should help keep down the incidence of failures in the system. While there is an ex ante sense in which inter-bank credit can play a stabilizing role several studies have emphasized the ex post destabilizing implications of one bank’s failure as the inter-bank credit system is susceptible to contagion. In an early paper, Iori et al. (2006) have shown that when banks are more heterogeneous in their characteristics (either in size or appetite for risk), increasing interbank connectivity initially decreases the probability of an individual bank default to occur. However, if defaults occur, they are more likely to initiate large default cascades. Thus, the relationship between the level of interconnectedness in the interbank markets and financial contagion is non-monotonic. Gai and Kapadia (2010) have further shown that increasing the connectivity of the banking network the system become more resilient to contagion triggered by the default of a random bank, but more fragile following the failure of highly connected nodes. A number of authors have explored the role of the interbank network structure on contagion (Nier et al. 2007; Karimi and Raddant 2016; Georg 2013; Krause and Giansante 2012; Lenzu and Tedeschi 2012) and compared how defaults propagate on scale-free, random, small world and core periphery networks under different modeling assumptions. Battiston et al. (2012) have developed a novel methodology to quantify the unrolling of distress between lenders and borrowers even before a borrower’s default, as creditors who are exposed to distressed debtors suffer a deterioration of their credit quality.

In addition to direct knock-on effects, the market impact of liquidating overlapping portfolios, in non-perfectly liquid markets, can amplify financial instabilities triggered by distressed banks. The liquidation pressure, typically driven by binding leverage constraints, can in fact lead to fire sales and create new contagion channels, as shown by Caccioli et al. (2014) and Aymanns and Farmer (2015). A third source of contagion has been identified in liquidity hoarding (Anand et al. 2013). A number of authors have in fact shown, using multi-layered networks, that the interaction of these different contagion channels can substantially amplify the effect of each individual one (Klimek et al. 2015; Montagna and Kok 2016; Covi et al. 2021). Multilayer networks have also been used to assess the impact of contagion when assets are disaggregated by seniority and/or maturity (see, e.g., Poledna et al. 2015; Hüser et al. 2018, 2019; Bargigli et al. 2015).

An increasing number of agent-based models have considered the interrelation between the financial market and the real economy, and explored the potential for ABMs to test the effectiveness of micro and macroprudential polices, such as Basel II and Basel III. Ashraf et al. (2017) have studied the role of loan-to-value ratios and static capital-adequacy regulation showing that less strict micro-prudential bank regulations allow the economy to recover faster from a crisis. Cincotti et al. (2012) have shown that lower capital-adequacy ratios can spur growth in the short run, but lead to more serious economic downturns in the long run as the number of bankruptcies of highly leveraged banks and firms grow, leading to credit rationing. Their simulations show that dynamic adjustment of capital requirements is generally more successful than fixed tight capital requirements in stabilizing the economy and improving the macroeconomic performance. Popoyan et al. (2017) and Krug et al. (2015) have shown that the components of Basel III are non-additive: the inclusion of an additional lever does not always improve the performance of the macroprudential regulation and their joint impact is more effective than the sum of their individual contributions. Assenza et al. (2018) have tested two macro-prudential policies, a modification of the maximum leverage ratio and the required liquidity ratio and shown that the former is more effective than the latter in terms of reducing the frequency of crises. However, no difference emergence as far as the duration of the crises is concerned. Riccetti et al. (2018) study the effect of minimum level of capital and lending concentration towards a single counterpart and support the introduction of regulatory rules, such as the Capital Conservation Buffer. Gurgone et al. (2018) allow banks to set endogenously their leverage and capital targets (within the bounds imposed by regulators) and as a result, when financial downturns occur, banks tend to amplify them by withholding liquidity from the interbank and credit markets and by seeking higher interest rates on the funds which they make available. This financial amplification mechanism (see also Delli Gatti et al. 2010) is exacerbated by the pro-cyclical effects of the prudential regulations.

Alternative resolution mechanisms of banking crises have been investigated by Klimek et al. (2015) who find that liquidation is the best policy during expansions, whereas bail-ins achieve better financial and economic stability during recessions. Poledna and Thurner (2016) have proposed the introduction of a tax on individual transactions, proportional to their marginal contribution to overall systemic risk. Their simulations demonstrate that such a Systemic Risk Tax leads to a self-organized restructuring of the financial network essentially eliminating the risk of banks’ collapsing. Notably, the restructuring occurs without loss of transaction volume and efficiency. On the contrary, when a Tobin tax or Basel III capital surcharges are imposed on systemically important financial institutions, the ABM leads to an increase of the cost of credit to the real economy.

Some authors, however, have shown that there, thus, appears to be no one-size-fits-all solution to financial regulation. For example Gabbi et al. (2015) have suggested that the effects of regulatory leverage ratios on the banking sector’s performance can vary in a complex and non-monotonic way with the state of the economy, the degree of connectivity of the interbank market and the amount of information available to market participants on bank risks while Riccetti et al. (2021) have shown that the effectiveness of the Basel III countercyclical capital buffer depends on the features of the business cycle.

Overall, these studies have shown that Agent-Based Models are powerful tools to understand the mechanism that lead to observed stylized fact in financial markets and to explain the unfolding of systemic risk in financial systems. By running a large number of simulations, changing the behavioral rules and the model parameters, ABMs can generate a rich set of data to evaluate the consequences of shocks, that can emerge endogenously or be imposed exogenously, and explore the effect of stabilization policies under counterfactual scenarios. Particularly for macro-finance applications, where data are scarce and experiments are limited, ABMs offer invaluable computational laboratories for evaluating what-if scenarios.

ABMs have so far mostly been used to generate insights and qualitative descriptions of scenario that may occur rather than quantitative forecasts. However, there have been some successful examples of forecasting with empirically calibrated financial agent-based models such as the work of Braun-Munzinger et al. (2018) on the corporate bonds markets. ABM simulation results can vary dramatically depending on which assumptions are used. As granular data sets of financial transactions are starting to be collected, it will become possible to test the realism of the behavioral assumptions and of the rules of interactions in the agent-based models. A careful calibration of these models to micro-level market data will enable the full potential of ABMs, as effective tools for assisting policy makers and market participants in their decision-making processes, to be exploited.

## Controlled laboratory experiments

Behavioral economics brings psychological foundations to economics aiming at better explaining economic phenomena. The emphasis of behavioral economics is basically on the effects that psychological, cognitive, emotional, cultural, and social factors have on individual as well as collective decision-making (see, e.g., Thaler 2016). Traditionally, behavioral economics has largely relied on evidence generated by controlled laboratory experiments with human subjects, where all those behavioral aspects are naturally considered (see, e.g., Smith 1989).

Contrary to the paradigm of rationality, experimental economics has shown that the heterogeneity of human subjects (e.g., different risk attitude, preferences or cultural background), their different degrees of bounded rationality and cognitive capabilities strongly influence their decisions. ABM builds upon a similar background, namely the pre-analytical vision that the assumption of heterogeneous interacting agents with different and given degrees of bounded rationality better captures micro-level properties of (macro) economic phenomena. ABM and experimental economics share, therefore, the departure from the representative rational optimizing agent as a fundamental building block for the analysis of economic phenomena. Whereas ABM assumes the heterogeneity of economic agents, controlled human subject experiments unavoidably deal with it. It is, thus, natural combining these two approaches, studying potential synergies and complementaries in dealing with the behavior and interaction of heterogeneous agents. Despite the long tradition of the experimental and ABM approaches to describe economic phenomena, it is only recently that several contribution consistently employed the findings of controlled experiments on the determinants of human behavior in the design of artificial agents in ABM. Fewer are, instead, the contributions of ABM in complementing experimental economics.

We claim that an interesting new literature has emerged, attempting to combine experimental and computational methodologies, thereby taking advantage of the synergies between them. Based on this literature in particular Duffy (2006) describes the common characteristics shared by ABM and controlled human subjects experiments: (1) a bottom-up modeling approach, contrary to top-down representative agent models, which naturally cope with heterogeneous agents; (2) complex interactions among agents, assuming that the aggregate behavior of interacting agents does not necessarily coincide with the behavior of the individual; and (3) agents which posses various degrees of bounded rationality. In their surveys, Arifovic and Duffy (2018) as well as Mauersberger and Nagel (2018) report several examples of laboratory experiments supported by computational simulations that show the crucial role of heterogeneity in describing the behavior of human subjects.

In this vein, Contini et al. (2006) list several examples of the complementarities between ABM and human subjects experiments. ABM can help explaining the behavior observed in human subject experiments and, at the same time, experimental data can be employed in calibrating and validating ABMs. When designing a laboratory experiment, a calibrated simulation can guide the experimentalist on the sensitivity of the subjects’ behavior to changes in the key parameters of the experimental design (see, e.g., Arifovic and Petersen 2017). Additionally, ABM simulations can be used for replicating human-based experiments using the experimental initial conditions, for increasing the number of periods and/or the number subjects, or for giving the opportunity to conduct a robustness test of the experimental findings (see, e.g., Hommes and Lux 2013). Conducting controlled laboratory experiments with human subjects imply the existence of budget and time constraints, that imposes limits to the number of participants (agents) and periods, that do not apply to ABM simulations.

Taking stock of that, however, we find that in most of the contributions, the combination of experimental and ABM simulations focused on explaining experimental data using ABM simulations, whereas we do not find many examples where experimental data served to complement the ABM findings. We think that one of the reason lies in the higher flexibility of computational agent-based models as compared to experimental settings, given the strong constrains in dealing with controlled human subjects experiments. Additionally, we should consider that nowadays ABMs have become much more complex than experimental settings, embracing large macro-simulations of the entire economy.

Despite their simplicity, controlled laboratory experiments allow for collecting data that in the real world are not available, like expectations formation or cognitive abilities or biases of human subjects that can be used to endow artificial agents in ABMs with more realistic characteristics and behavior following, for example, adapting learning rules.

## Estimation of agent-based models

Agent-based models have been developed for different purposes. Historically, some of the first examples of disaggregated models of economic systems have been microsimulations (pioneered, e.g., by Orcutt 1957; Orcutt et al. 1961) that were mainly developed as decision support system for economic policy. While these models are usually carefully calibrated using empirical distributions of agents’ characteristics (such as the age structure of a population to forecast the development of pension expenditures), they have not been subject to rigorous econometric validations. Indeed, the idea of estimation seems alien to this class of models as they are dominated by both institutional detail and a close mapping of certain empirical attributes of the population that are deemed important for a certain type of policy question.^4^ There are typically few behavioral relationships and those that exist are well-represented by statistical averages over the large underlying populations (e.g., retirement age, divorce rates, etc.). In contrast, the more recent branch of theoretically motivated ABMs that emerged since the 1990s have a different relationship with data: with few exceptions, the motivation of these ABMs has been the desire to explain via behavioral assumptions certain stylized facts that more aggregate, traditional models had left unexplained. The guiding idea of this literature is that certain salient features of our economic reality can only be explained as the outcome of a process of self-organization of the activity of a large ensemble of interacting, heterogeneous agents (see, e.g., Gallegati and Kirman 2012). The first brand of such models has mainly addressed the well-known but mysterious stylized facts of financial markets such as the particular broad distribution of returns (fat tails) and the extremely large correlation in all measures of their range of fluctuations (clustered volatility), see also Lux (2009b).

Slightly later, a related literature on macroeconomic ABMs has been developed (e.g., Dawid and Delli Gatti 2018) which addresses macroeconomic stylized facts such as the distribution of booms and recessions, and cross-correlations between key macroeconomic variables. Other areas of intense ABM research include industrial dynamics (e.g., Axtell 2018), and the emergence of stratified distributions of income and wealth (Chakraborti 2011). With the orientation at measurable stylized facts, empirical validation and estimation of their parameters should be a top priority of the ABM community. Indeed, the justification of the relatively heavy apparatus of models with a multitude (or at least multiple groups) of agents rests on its capacity to explain data better than traditional approaches using structural equations without micro foundations, or the representative agent models that have been particularly popular in macroeconomics. In some areas, it seems easy to score as goal for ABMs as, for instance, important and well-documented regularities such as the size distribution of firms and the Pareto-type distribution of income and wealth defy any attempt of their explanation without disaggregated agents. Other stylized facts like those of financial data had in the pre-ABM literature only be explained in a tautological way: if returns are fat tailed and come with clustered volatility, so must have been the distribution of news on which they are based. More demanding is the task in macroeconomics where there exist well-established models at least for the cross-sectional patterns characteristic of business cycles (although the performance of the traditional DSGE models is not really considered satisfactory, see also Stiglitz 2018).

Early literature on the validation of ABMs has mostly focused on calibration rather than rigorous parameter estimation. Often researchers have expressed concerns whether estimation in the usual understanding of the term should be possible at all for ABMs (e.g., Fagiolo et al. 2007a). Such a concern might indeed be valid for the large early microsimulation models built for economic policy and their present-day descendants. However, for most of the more theoretically motivated ABMs and also the heterogeneous agent models with only a few groups of agents, rigorous estimation is indeed methodologically straightforward, yet practically often difficult. In terms of statistical methodology, the possibility of identification of parameters is guaranteed because most ABMs as they exist are Markov processes (a fact already emphasized by Aoki 1998). The nonlinearities inherent in an ABM framework also typically guarantee that problems such as collinearity are not an issue, at least in principle. However, the proliferation of parameters in many ABMs can easily lead to near-collinearity or parameters, that fail to exert much influence on any statistic used in an estimation algorithm (see the experiments in Lux and Zwinkels 2018). Rigorous estimation should, therefore, be a most welcome device to impose discipline on ABM modeling, and estimation results should be brought to good use in model development (e.g., when irrelevant parameters are encountered in an estimation).

The focus on stylized facts as a motivation to develop ABMs in the first place suggests an empirical approach that uses the available knowledge on interesting statistics of the data: this has often made the generalized Method of Moments (GMM) or Simulated Method of Moments (SMM) the methodology of choice.^5^ Examples include Jang (2015), Grazzini and Richiardi (2015), Chen and Lux (2018) or Franke and Westerhoff (2012). Simulation-based estimation seems to suggest itself since the explanatory power of ABMs is mostly explored via Monte Carlo simulations anyway. GMM and SMM also dispense with the necessity of a closed-form solution or numerical approximation for the likelihood which is almost never available in ABMs (an exception is the model estimated in Lux 2009a, 2012).^6^ The major drawback of GMM/SMM is a much lower efficiency of the resulting estimates than under a maximum likelihood approach. If the likelihood can be formulated but not solved explicitly, stochastic approximations of the likelihood via a sequential Monte Carlo algorithm or particle filter would be a possibility (see also Lux 2018). In this approach, a swarm of candidate parameter vectors is updated through the iterated computation of their likelihood values via importance sampling and the averaging over the active particles in each time step provides the approximation of the likelihood function. Again, this approach is computation intensive as it uses simulations of a large number of replications of the model (with different parameter values), but it provides a higher efficiency of the so attained parameter estimates than GMM/SMM. Since in this framework, the ABM is interpreted as a state-space model with both hidden variables and measurable variables, another advantage is that the particle filter allows to identify the dynamic evolution of hidden variables. These could be the distribution of expectations, strategies or attributes among agents, and would often be of immediate economic interest. Sequential Monte Carlo can be used in frequentist estimation as well as in a Bayesian context (see also Grazzini et al. 2017; Berschinger and Mozzhorin 2020; Lux 2020).

## Outlook and future directions

There are numerous promising avenues for research on agent-based models, some have already been touched upon in the previous sections. A particular strength of ABM has always been its flexibility towards the application to new problems. While certain classes of models have been established in fields like macroeconomics or financial markets, ABM has always been a transdisciplinary methodology that can be adapted to problems with different rules, interaction mechanisms and behavioral phenomena.

A current example is the data-driven models that have been developed for the COVID-19 pandemic. Here, ABMs can be an effective tool to model human interactions and disease dynamics over space and time and offer realistic predictions in terms of the scale of an outbreak or the effectiveness of different interventions (Goldstein et al. 2020; Squazzoni et al. 2020; Lux 2021).

ABMs can also be used to study problems that result from the increased use of AI, for example the societal impact of ranking algorithms, recommender systems and its possible reinforcements of social inequalities and biases. In situations in which the given data is noisy or biased, ABMs can be used to generate priors to produce scenarios for machine learning algorithms in a semi-supervised manner to reduce errors and prevent the amplification of distortions. Also, once artificial agents have been designed based on the behavior of human subjects, they can be implemented in large-scale simulators (see Dosi et al. 2020). Such synergy between ABM and experimental methodology is at its infancy and, in our opinion, constitutes an exciting avenue of future research.

Further research is also needed on the estimation of ABMs, since not too much is known about the pros and cons of different methods. Available models have mostly allowed for at least the formulation and stochastic approximation of a likelihood function. When models become more complex, such approximations will often not be feasible anymore. In such cases, a promising tool—besides GMM/SMM—should be Approximate Bayesian Computation (ABC). This framework uses measurements (moments) of the data other than the likelihood (Sisson et al. 2005; Toni et al. 2008), and allows to approximate the posterior distribution of the parameters via a rejection sampling or Markov Chain Monte Carlo algorithm. While this approach has become very popular in ABMs in ecology (see Csilléry et al. 2010), economic applications are absent so far.

## Footnotes

1 In the following, we use ABM as the abbreviation for agent-based model and agent-based modeling.

2 For a formal introduction see Wilensky and Rand (2015) and Gilbert (2008).

3 Further important research outside the scope of this overview has been done by analyzing supply chains and logistics as well as by applying Game Theory to models of network formation.

4 The *International Journal of Microsimulation* might be consulted for an overview over this rich universe of agent-based models for policy applications that almost constitutes a parallel world to the more theoretical ABMs developed in academia.

5 A more complete review of estimation techniques for ABMs can be found in Lux and Zwinkels (2018).

6 Another type of simulation-based approach is pursued by Recchioni et al. (2015): rather than fitting moments they fit a financial market model with two groups to the entire historical trajectory of some stock market indices. Since they take the deterministic ’skeleton’ of the model, and do not specify any error term, their approach does neither follow the usual principle of minimum distance nor maximum likelihood estimators.
